# Are Accelerated and Enhanced Wave Function Methods Accurate to Compute Static Linear and Nonlinear Optical Properties?

**DOI:** 10.1021/acs.jctc.2c01212

**Published:** 2023-03-02

**Authors:** Carmelo Naim, Pau Besalú-Sala, Robert Zaleśny, Josep M. Luis, Frédéric Castet, Eduard Matito

**Affiliations:** † Donostia International Physics Center (DIPC), Manuel Lardizabal Ibilbidea 4, 20018 Donostia, Euskadi, Spain; ‡ Univ. Bordeaux, CNRS, Bordeaux INP, ISM, UMR 5255, F-33400 Talence, France; ¶ Polimero eta Material Aurreratuak: Fisika, Kimika eta Teknologia, Kimika Fakultatea, Euskal Herriko Unibertsitatea UPV/EHU, 20080 Donostia, Euskadi, Spain; ∥ Institut de Química Computacional i Catàlisi and Departament de Química, Universitat de Girona, 17003 Girona, Catalonia, Spain; ⊥ Faculty of Chemistry, Wrocław University of Science and Technology, Wyb. Wyspiańskiego 27, PL−50370 Wrocław, Poland; # Ikerbasque Foundation for Science, 48011 Bilbao, Euskadi, Spain

## Abstract

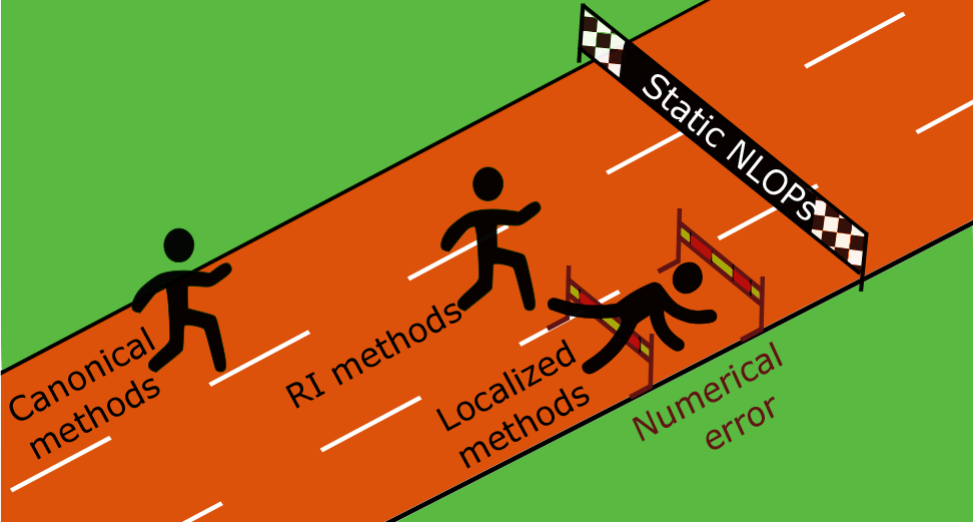

Key components of organic-based electro-optic devices are challenging to design or optimize because they exhibit nonlinear optical responses, which are difficult to model or rationalize. Computational chemistry furnishes the tools to investigate extensive collections of molecules in the quest for target compounds. Among the electronic structure methods that provide static nonlinear optical properties (SNLOPs), density functional approximations (DFAs) are often preferred because of their low cost/accuracy ratio. However, the accuracy of the SNLOPs critically depends on the amount of exact exchange and electron correlation included in the DFA, precluding the reliable calculation of many molecular systems. In this scenario, wave function methods such as MP2, CCSD, and CCSD(T) constitute a reliable alternative to compute SNLOPs. Unfortunately, the computational cost of these methods significantly restricts the size of molecules to study, a limitation that hampers the identification of molecules with significant nonlinear optical responses. This paper analyzes various flavors and alternatives to MP2, CCSD, and CCSD(T) methods that either drastically reduce the computational cost or improve their performance but were scarcely and unsystematically employed to compute SNLOPs. In particular, we have tested RI-MP2, RIJK-MP2, RIJCOSX-MP2 (with GridX2 and GridX4 setups), LMP2, SCS-MP2, SOS-MP2, DLPNO-MP2, LNO-CCSD, LNO-CCSD(T), DLPNO-CCSD, DLPNO-CCSD(T0), and DLPNO-CCSD(T1). Our results indicate that all these methods can be safely employed to calculate the dipole moment and the polarizability with average relative errors below 5% with respect to CCSD(T). On the other hand, the calculation of higher-order properties represents a challenge for LNO and DLPNO methods, which present severe numerical instabilities in computing the single-point field-dependent energies. RI-MP2, RIJK-MP2, or RIJCOSX-MP2 are cost-effective methods to compute first and second hyperpolarizabilities with a marginal average error with respect to canonical MP2 (up to 5% for β and up to 11% for γ). More accurate hyperpolarizabilities can be obtained with DLPNO-CCSD(T1); however, this method cannot be employed to obtain reliable second hyperpolarizabilities. These results open the way to obtain accurate nonlinear optical properties at a computational cost that can compete with current DFAs.

## 1 Introduction

In several fields as different as molecular biology or material science, the demand for functional materials bearing specific electro-optical features is increasing yearly,^1−3^ for instance, in the construction of two-photon absorption or noninvasive three-dimensional fluorescence microscopy devices.^4,5^ The key compounds used for building such devices are, however, difficult to design or optimize since most of the newest applications are based on the nonlinear response of these molecular units upon interaction with light, which is a physical process difficult to model or rationalize.

The energy of a molecule subjected to an external static electric field **F** can be expressed as a Taylor expansion of its unperturbed energy, *E*_0_, with respect to **F**:
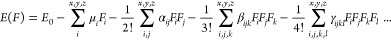
1The expansion coefficients in eq 1 are, respectively, the components of the dipole moment μ_*i*_, polarizability α_*ij*_, first hyperpolarizabilty β_*ijk*_, and second hyperpolarizability γ_*ijkl*_ tensors, which can be expressed as consecutive derivatives of the energy with respected to *F*_*i*_ calculated at *F*_*i*_ = 0. Considering electric fields applied along the *z* direction (*F*_*i*_ = *F*_*z*_), the corresponding diagonal components of the tensors are
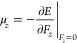
2
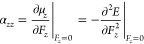
3
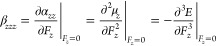
4
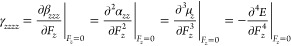
5These quantities describe the magnitude of the static linear and nonlinear responses of the chemical system to an external electric field; hence their accurate computation is crucial for the bottom-up design of optic, electro-optic, and optoelectronic devices. Despite the broad scope of application of density functional approximations (DFAs), these methods often struggle at reproducing static linear and nonlinear optical responses of molecular systems. Some DFAs (usually implying hybrid exchange-correlation functionals with a large percentage of Hartree–Fock exchange) can reproduce the correct trends in the evolution of properties within a series of molecules, although they often fail to accurately reproduce the magnitude of the electrical nonlinear response properties.^6−10^ At the heart of this problem is the delocalization error,^11^ inducing the overdelocalization of electrons, which also leads to the underestimation of reaction barriers and charge-transfer excitation energies and rate-constants,^11,12^ the overestimation of the conductance of molecular junctions, the magnetizability of strong antiaromatic molecules,^13^ electron conjugation,^14^ and aromaticity.^15−20^ A necessary condition to avoid the consequences of the delocalization error on electrical responses is the correct asymptotic decay of the exchange-correlation potentials.^21,22^ The latter is easily imposed using a range-separated (RS) DFA. However, even state-of-the-art DFAs using optimally tuned range-separation parameters sometimes incorrectly reproduce the magnitude of β and γ for relatively simple molecules.^7,23^ Even though the delocalization error is often the main problem in DFAs, electron correlation (beyond the local or semilocal approximations included in most DFAs) is also an essential factor to consider. Indeed, double hybrids often improve the performance of their hybrid or range-separated peers for computing nonlinear optical (NLO) properties.^24^ In addition, some of us have recently unveiled that most DFAs suffer from spurious oscillations that affect the calculation of high energy/property derivatives with respect to nuclear coordinates, which contribute to the static NLO properties (SNLOPs).^6,25^

On the other hand, wave function methods (WFMs) are exempt from many problems of DFAs, in particular, from the delocalization error. The hierarchical structure of WFMs, such as configuration interaction (CI), Møller–Plesset perturbation theory, or coupled-cluster (CC), provides a systematic way toward the exact solution for a given atomic basis set. In the framework of density functional theory (DFT), Perdew defined the Jacob ladder, which gives a qualitative indication of the expected accuracy of a DFA according to its type; unfortunately, these expectations are not always met for SNLOPs.^6,26^ High-order WFMs are often considered more accurate than DFAs. In particular, the CC method including single and double excitations with a perturbative estimation of triples [CCSD(T)]^27^ is often regarded as the gold standard of WFMs. The computational time of canonical CCSD(T) single-point energy calculation scales as , where *N* is the number of electrons and *M* is the number of basis functions of the system. Hence, despite the advantages of WFMs over DFAs, the computational cost of the former usually prevents the calculation of SNLOPs beyond cost-effective methods such as the second-order Møller–Plesset perturbation theory (MP2).^24^ However, the development of the linear response formalism of CC^28,29^ method and some seminal papers on small molecules^30,31^ using wave function methods are worth highlighting. Besides, MP2 still presents an unfavorable scaling () compared to most DFAs (excluding DFAs from the fifth rung such as the double hybrids) and lacks the accuracy to compete with CCSD(T) in a number of situations.^32−34^

Many attempts have been made to increase the cost-efficiency of WFMs.^35−41^ They can be classified into two groups: techniques developed to bring down the computational cost of WFMs (accelerated WFMs) and methods aiming at increasing the accuracy of the low-cost WFMs (enhanced WFMs). Among the available acceleration techniques, resolution of identity (RI)^42,43^ approximations have become of routine use in many WFMs, the most popular being RI-MP and RI-CC methods.^35,44^ RI techniques have also been introduced for Hartree–Fock (HF) and DFT methods.^45−47^ These methods show excellent performance in calculating energies, with considerable time savings.^48−51^ Other methods are based on orbital localization, exploiting the local nature of dynamic correlation.^52^ They are usually coupled with RI approximations, and by localizing natural orbitals they can drastically reduce the computational cost and reach an almost linear scaling with the size of the system, i.e., .^53−58^ Enhancement techniques exploit some of the systematic deficiencies of WFMs. For instance, MP2 underestimates the opposite-spin (OS) correlation, which is unbalanced with respect to the amount of same-spin (SS) correlation because it is based on the Hartree–Fock wave function, which considers the Pauli principle but treats OS pairs as statistically independent pairs. One way to compensate for it is to introduce variable amounts of SS and OS MP2 correlation in what is known as the spin-component scaled MP2 (SCS-MP2) method.^59^

Benchmark studies of thermodynamics, kinetics, and some molecular properties have been performed on accelerated and enhanced methods.^60−63^ However, thus far, a systematic study of the performance of these methods for computing static NLO properties is missing in the literature. In this work, we assess the accuracy and computational cost of several enhanced and accelerated techniques applied to CCSD, CCSD(T), and MP2 methods against their canonical counterparts, focusing on the calculation of dipole moments, polarizabilities, and first and second hyperpolarizabilities.

## 2 Methodology

### 2.1 Theoretical Methods

**Figure 1:**
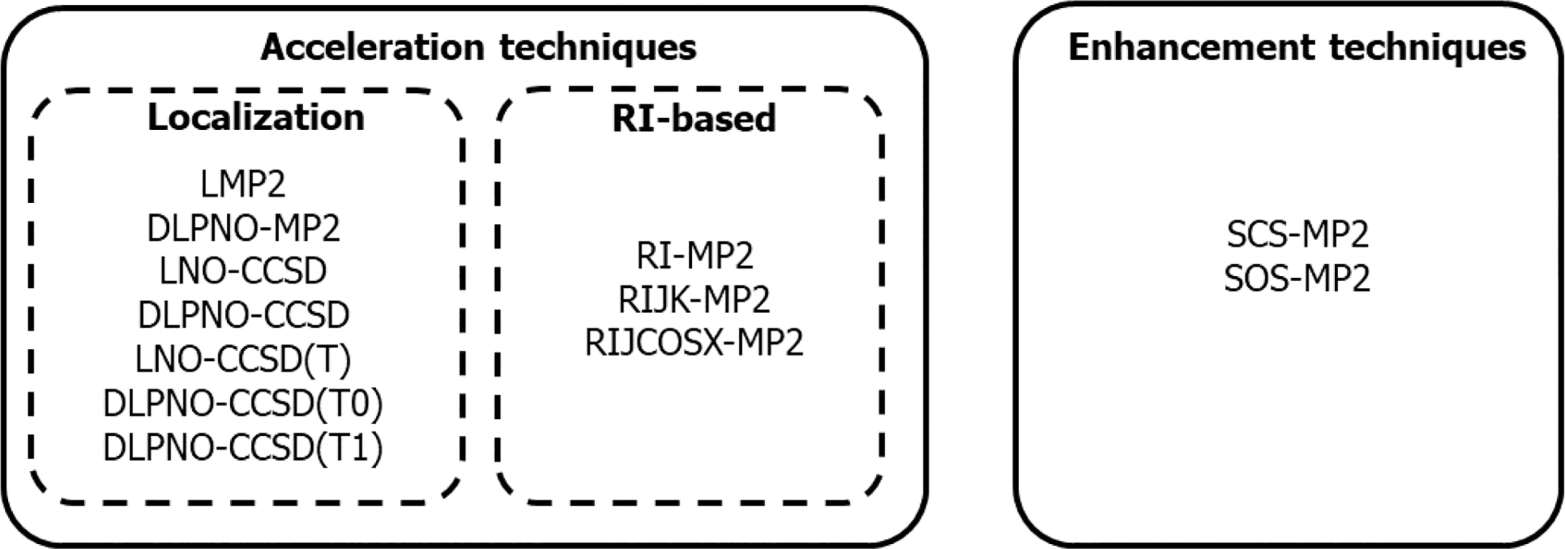
Summary of the methods studied in this work.

In this section, we briefly review various accelerated and enhanced WFMs. Acceleration techniques aim to reduce the canonical (unaccelerated) method’s computational time without sacrificing accuracy. On the other hand, enhancement techniques aim at improving the accuracy of the canonical method without a significant increase of the computational cost. A summary of the methods considered in this study is provided in Figure 1.

#### 2.1.1 Accelerated Methods

Among acceleration techniques, one of the most popular is the resolution of identity (RI). Within this scheme, the two-electron (four-index) integrals, (*ab*|*cd*), are approximated as two- or three-index integrals, thus reducing the scaling with respect to the basis set size. In particular, orbital products are expanded into an auxiliary basis of functions χ_*B*_ in a density fitting process:
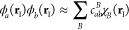
6where the error integral is defined as
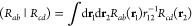
7with
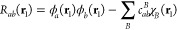
8The coefficients, *c*_*ab*_^*B*^, are determined by minimizing the error integral:
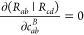
9which implies that every *R*_*ab*_ is orthogonal to every auxiliary basis function χ_*B*_, giving rise to the three-center integrals (*ab*|*B*) that approximate the two-electron integrals:
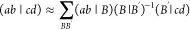
10where all previous integrals, including those with only two indices, contain the 1/*r*_12_ operator. The main limitation of the RI method is that the density fitting has to be parametrized for a specific canonical basis set,^64,65^ and auxiliary basis sets are not available for all basis sets reported in the literature. Implementations of RI for MP2, CC, or DFT are available. Unlike MP2, in CCSD the limiting step of the calculation is not the integral transformation; hence, one cannot expect as large savings with RI as in MP2. In practice, the RI-CCSD calculation is significantly slower than the canonical one in many computational packages. For instance, in ORCA, there are some disk space savings, but the computationally dominant steps are executed less efficiently with RI.^66^ For this reason, in this paper, we have limited our assessment to RI-MP2 variants.

The RI approximation can be applied independently to the self-consistent field (SCF) part of the calculation, to the post-HF, or to both. In the present work, we applied the RI either to the MP2 part only^67−69^ (RI-MP2, hereafter) or to both the SCF and the MP2 parts. Two different versions of RI-SCF calculations have been tested, the RI-JK-SCF method,^70^ in which both the Coulomb (J) and exchange (K) integrals are treated with the RI method, and the *chain of spheres* method (RI-J-COSX-SCF), in which the Coulomb part is computed with the RI approximation and the exchange part is computed by numerical integration over a predefined grid.^71^ Both methods are implemented in ORCA,^66^ and we refer to them throughout the paper as RIJK-MP2 and RIJCOSX-MP2, respectively. Additionally, we have tested a tighter COSX grid (GridX4 in ORCA 4.0 input) referred to as COSX2 in this manuscript.^72^

The second family of acceleration techniques tested in this work is designed to take advantage from the eminently local character of electron correlation.^52^ In this regard, the transformation from canonical to localized orbitals can be achieved by a unitary transformation of the wave function. Depending on the constraints added to the unitary transformation, several localization schemes may arise. Recently, methods based on the localized pair natural orbitals (LPNOs) are becoming very popular as they introduce a drastic reduction of the computational cost, resulting in an almost-linear scaling with the molecular size. These methods employ the pair natural orbitals (PNOs) formulation, reducing the virtual space of the calculation^73^ by localizing its orbitals through the Foster–Boys algorithm,^74,75^ which consists in minimizing ⟨*L̂*⟩ (with *L̂* = |*r⃗*_1_ – *r⃗*_2_|^2^). Other important localization schemes used in alternative contexts are the Edmiston–Rudenberg^76^ or the Pipek–Mezey^77^ ones, which impose the minimization of the orbital self-repulsion and the atomic Mulliken charges, respectively. It is important to distinguish methods that localize the orbitals after the SCF calculations from those like the Extremely Localized Molecular Orbitals (ELMO)^78^ scheme, which applies directly the variational principle to the constrained many-body Slater determinant.

Among the localization methods available in the literature, we decided to test two different schemes for the calculation of the nonlinear optical properties. The first one is the domain localized pair natural orbital (DLPNO) method^54,55,58^ implemented in ORCA.^66^ The second one is the localized natural orbital method (LNO) developed by Kállay and co-workers^79^ implemented in the MRCC package.^80^ These two methodologies use different strategies to construct the virtual domain. The machinery behind DLPNO is rather convoluted and can be summarized as follows. A set of pair natural orbitals (PNOs) providing the most compact description of the virtual space is constructed. The latter PNOs are obtained through the diagonalization of the pair density matrix for every pair of localized occupied orbitals. Finally, the DLPNO method expands the PNOs in terms of certain basis functions, more specifically, into the set of Pulay’s projected atomic orbitals (PAOs),^52^ belonging to a specific electron-pair domain.^37^ Alternatively, the LNO method first localizes the MOs using a distance criterion. Subsequently, each localized MO is assigned to a local subspace of occupied and virtual orbitals, which is constructed from approximate Møller–Plesset frozen natural orbitals. Finally, the CC equations are solved for each LNO subspace, and the total correlation energy is obtained from the summation over all the subspaces. The main difference between DLPNO and LNO schemes is that the former defines the interacting subspaces from electron pairs, while the latter uses individual electrons.^56,80−82^

Such definitions for the localized orbitals (either LNO or DLPNO) can be used to efficiently compute post-HF energies and wave functions. In this manuscript, we have assessed LMP2,^40,57^ which uses the LNO localization method, LNO-CCSD,^57,80^ DLPNO-MP2,^54^ and DLPNO-CCSD in computing electric properties.^37^ We also computed analytic DLPNO-MP2 dipole moments and polarizabilities using ORCA 5.0.^62,83^ We refer to this method as DLPNO-MP2-α. We compared DLPNO-MP2-α analytic polarizabilities with numerical DLPNO-MP2 polarizabilities (obtained from energy derivatives, see eq 3) to evaluate the magnitude of the numerical errors in the calculation of polarizabilities using the Rutishauser–Romberg technique (see below). We also considered triple perturbative corrections to DLPNO-CCSD using the two alternative approximations available: DLPNO-CCSD(T0),^53^ in which the triple-excitation corrections are calculated following the semicanonical approximation, and DLPNO-CCSD(T1),^58^ which is more expensive (and considered more accurate) because it is partially iterative. The triple perturbative corrections have been also tested for the LNO scheme, referred as LNO-CCSD(T).^79,84^

#### 2.1.2 Enhancement Methods

Regarding the enhancement methodology, we assessed the spin-component scaled MP2 method. SCS-MP2 does not reduce the computational time explicitly, but it effectively improves the quality of the results of a canonical MP2 calculation by increasing the amount of opposite-spin (OS) correlation and scaling down same-spin (SS) correlation,
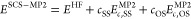
11where *c*_OS_ = 6/5 and *c*_SS_ = 1/3 for Grimme’s SCS-MP2 (as opposed to canonical MP2, where *c*_OS_ = 1 and *c*_SS_ = 1).^85^ Head-Gordon and co-workers suggested a scaled opposite-spin MP2 (SOS-MP2), which takes values *c*_OS_ = 1.3 and *c*_SS_ = 0. By excluding the same-spin correlation, the computational complexity might be reduced from fifth to fourth order.^86^

### 2.2 Computational Details

Single-point calculations have been performed using an energy threshold of 10^–9^ a.u. for convergence of both the SCF and CC calculations. A tighter convergence criterion (10^–14^ a.u.) was also tested; however, the results for the (hyper)polarizabilities showed no significant improvement, whereas medium/large systems showed hampered convergence. All calculations have been performed with aug-cc-pVDZ in conjunction with the corresponding auxiliary basis set when needed. We did additional calculations at the RI-MP2 level using larger reoptimized auxiliary basis sets. As expected, the larger auxiliary basis sets reduced the errors of single-point energy calculations. However, we did not observe a systematic improvement in the SNLOPs, partly due to a larger numerical instability of differentiation of the single-point energies. We thus recommend using the default compact auxiliary basis recommended by ORCA developers (corresponding to the aug-cc-pVDZ auxiliary basis and including an additional contraction scheme for s- and p-block atoms) to compute SNLOPs using RI-MP2 methods.

Dipole moments, static linear polarizabilities, and first and second hyperpolarizabilities have been evaluated numerically through finite-field central derivatives of the total energy. These calculations have been performed on the range of external electric fields ±2^*j*^ × 10^–4^ a.u. with *j* = [0, 9] (1 a.u. = 51.422 V·Å^–1^). By construction, the finite-field central derivatives remove the truncation error caused by the higher-order terms of the Taylor expansion of the field-dependent energy with different parities of the derivative evaluated. In order to reduce the truncation error coming from neglecting the latter higher-order terms with the same parity of the derivative evaluated, the Rutishauser–Romberg (RR) formula has been employed,^87,88^
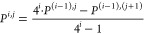
12where *P* is the calculated property, *i* is the RR iteration number, and *j* is the exponent entering the expression of the electric field amplitude (±2^*j*^ × 10^–4^ a.u.). In order to choose the *i* and *j* values minimizing the truncation error, the minimum of the difference between the *j*th and (*j* + 1)th rows for the same *i*th column of the matrix *P*^*i*,*j*^ is evaluated and defined as the Romberg Error (*RE*), namely,
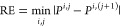
13

In order to verify which methods are numerically stable, we reported the Mean Absolute Romberg Error (MARoE) of each property and the relative MARoE (%MARoE) calculated by dividing MARoE by the average value. We observed that for systems with %MARoE higher than 25% and the optimal selected RR iteration of 1, the RR procedure tends to amplify the numerical errors instead of decreasing them. In these cases, the Romberg procedure was not used. Instead, the smallest finite-field central derivative of the rows that present the minimum difference was selected.

The localization schemes depend on a wide range of cutoffs, thresholds, and parameters that control the accuracy of the energy calculations and, subsequently, their derivatives.^61^ The developers of the DLPNO method identified three different sets of thresholds for the localization schemes, associated with a particular computational cost and accuracy. They employ the keywords LoosePNO, NormalPNO, and TightPNO to refer to these sets.^89^ At the same time, LNO developers identified three sets of parameters controlled by the variable *lcorthr* in the MRCC input.^90^ After a few tests, it became obvious that to reduce the numerical error associated with each single-point calculation, TightPNO should be used for DLPNO calculations and *lcorthr* = *VeryTight* for LNO. Calculations with looser cuttoffs are included in the Supporting Information for comparison. As an illustrative example, the relative errors committed by the numerical differentiation (estimated by %MARoE) of α, β, and γ applying several thresholds at the DLPNO-CCSD(T) level of theory for the γ-NLO set (see below) are summarized in Table 1.

**Table 1:** Relative Mean Absolute Romberg Error (%MARoE) on α, β, and γ Values Obtained from the Numerical Derivatives of the Energy, Calculated for Molecules of the γ-NLO Set (See Next Section) by Using Various DLPNO Methods Employing Different Thresholds for the SCF/CC Equations and Orbital Localization Scheme

			%MARoE		
Method	SCF/CC	Localization	α	β	γ
DLPNO-CCSD(T0)	VeryTight	NormalPNO	1	29	66
DLPNO-CCSD(T0)	VeryTight	TightPNO	0	24	60
DLPNO-CCSD(T0)	ExtremeSCF	TightPNO	0	22	52
DLPNO-CCSD(T1)	VeryTight	NormalPNO	0	31	77
DLPNO-CCSD(T1)	VeryTight	TightPNO	0	17	41

Improving the SCF/CC convergence from the VeryTight to the ExtremeSCF criterion in ORCA provides comparable %MARoE for α, β, and γ values. Therefore, considering the computational cost of using ExtremeSCF, we opted for the former criterion. The numerical derivative errors also show a large dependency on the localization cutoffs. In order to minimize these errors, we employed the highest TightPNO criterion. We also tested user-tailored combinations of the set of parameters, seeking an increase of accuracy for SNLOP calculations, but we did not find a situation where one particular parameter was singled out as the most relevant or dominant to improve the quality of the SNLOPs. In practice, the errors increase significantly with the order of the energy derivatives; hence, β and γ require the tightest criteria. Notice that in some cases, the numerical instability of the energies is so large that the error committed can exceed %MARoE = 95%. These conclusions about the need of high localization cutoffs are in line with the findings of Alonso, Martin, and co-workers,^61^ who identified that very tight cuttoffs for DLPNO are needed to reproduce the relative energy of extended porphyrins, as well as to study of weakly bound supramolecular complexes.^91^

The performance of enhanced and accelerated CCSD(T), CCSD, and MP2 WFMs has been assessed by comparison to reference values obtained using the corresponding canonical methods, by considering four statistical measures: the Mean Absolute Error (MAE), the Root Mean-Square Error (RMSE), the Maximum Error (MAX), and the percentage MAE (%MAE)
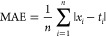
14
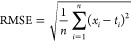
15
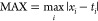
16
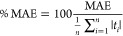
17where *t*_*i*_ and *x*_*i*_ are the reference and the predicted values for system *i*, respectively. The latter four indicators measure the *accuracy* of SNLOPs, which critically depends on the *precision* of the field-dependent single-point energies employed to perform the corresponding numerical derivatives. The energy precision is assessed through %MARoE, included in the Supporting Information.

### 2.3 Benchmark Sets

**Figure 2:**
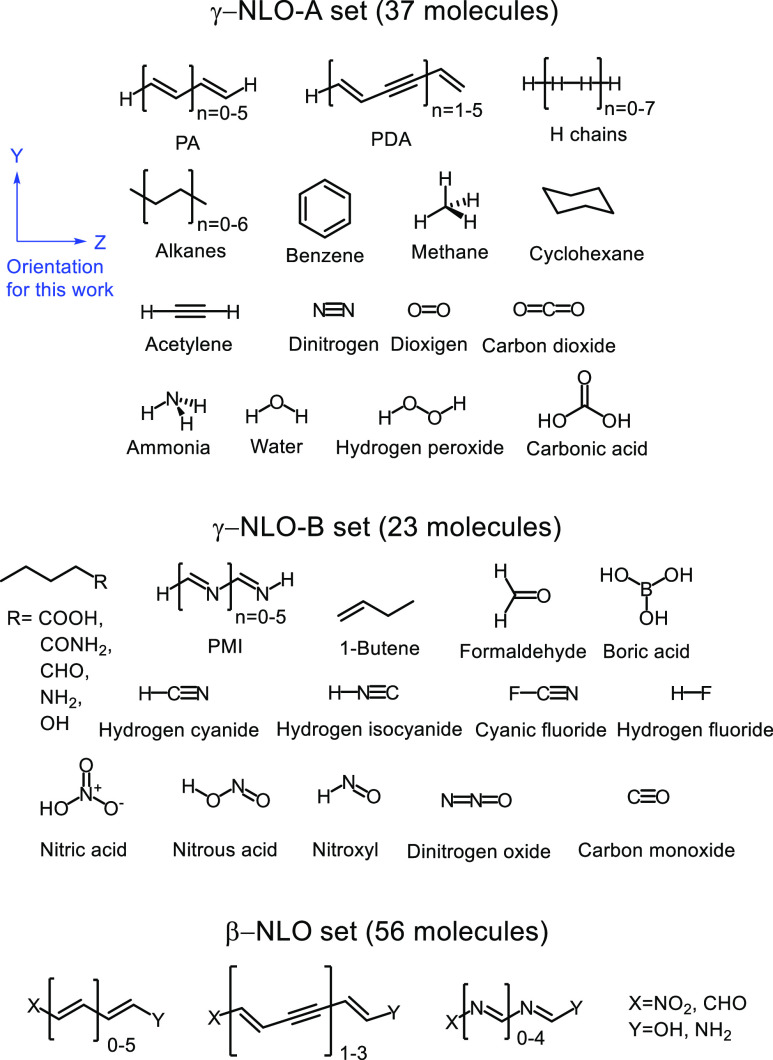
Benchmark γ- and β-NLO sets studied in this work. The subset γ-NLO-A contains molecules that, on the adopted orientation, are symmetric along the *z*-axis, while the subset γ-NLO-B contains molecules that are not symmetric along the *z*-axis.

Two benchmark sets are used in this work: the γ-NLO set^7^ and the β-NLO set (see Figure 2). The γ-NLO set contains 60 molecules, formed by 2 to 36 atoms of the second period and/or hydrogen. The latter set can be split into two subsets: the first one (γ-NLO-A, 37 molecules) contains molecules that, for the adopted orientation, are symmetric along the *z*-axis, while the second set (γ-NLO-B, 23 molecules) includes polar molecules oriented aligning their inertia axis to the *z* axis, and thus they are not symmetric along *z*. The γ-NLO-A set includes the first oligomers of two series of well-known NLO compounds—the all-trans polyacetylene (PA) and the polydiacetilene (PDA)—as well as some small organic and inorganic molecules, and weakly interacting H_2_ chains, which are particularly challenging systems for the computation of second hyperpolarizabilities.^92^ This set has been only employed for the evaluation of α_*zz*_ and γ_*zzzz*_ (even derivatives with respect to the electric field) because these molecules present, due to symmetry, null μ_*z*_ and β_*zzz*_ (odd energy derivatives). Conversely, the γ-NLO-B has been employed to evaluate μ_*z*_, α_*zz*_, β_*zzz*_, and γ_*zzzz*_. This set includes the first six oligomers of all-trans polymethineimine (PMI), the SNLOP calculation of which proves difficult for electronic structure methods.

The β-NLO set contains molecules with expected large β and γ. In particular, it consists of 56 π-conjugated push–pull systems that result from the functionalization of the terminal positions of PA_1–6_, PDA_1–3_, and PMI_1–5_ oligomers with two electron-withdrawing (−NO_2_, −CHO) and two electron-donor (−NH_2_, −OH) substituents.

Molecular geometries for both sets are available for public use (https://www.iochem-bd.org/handle/10/247254), and the dipole moment, polarizability, and hyperpolarizabilities for all methods assessed, as well as for reference CCSD(T), can be accessed through a separate file (see SI). Both data sets are also available at https://molprolab.com.

All the molecules have a singlet ground state, with the exception of O_2_, which presents a triplet state. Unrestricted calculations with localized methods are still not implemented in MRCC; therefore, O_2_ was excluded from this study.

The single-reference character of the molecules was assessed through a series of multireferences diagnostic criteria. On one side, we computed D1,^93^ D2,^94^ and T1^95−97^ diagnostics of the CCSD wave functions. We employed various natural-occupancy-based diagnostics for MP2 calculations, namely, NON, V, MRI,^98^ and I_ND_.^99−102^ According to the latter diagnostics, none of the molecules presents a very high multiconfigurational character (see Tables S7–S9), and therefore, single-reference coupled-cluster and MP2 methods are adequate to assess the electronic structure of such systems. Only the longitudinal components of the dipole moment vector (μ_*z*_) and of the polarizability (α_*zz*_) and hyperpolarizability tensors (β_*zzz*_ and γ_*zzzz*_) were computed for all the molecules (see Figure 2). For simplicity, hereafter the indices will be dropped and the diagonal tensor components will be noted μ, α, β, and γ.

## 3 Results

In this section, we will only show statistical errors with respect to some reference values. Absolute magnitudes of the static linear and nonlinear optical properties are available in the Supporting Information. The results are organized as follows: The performance of accelerated methods are first checked against their canonical counterparts. Then, we consider their accuracy by comparing the computed static optical responses with CCSD(T)/aug-cc-pVDZ reference values. Finally, we assess enhanced wave function methods.

### 3.1 Relative Performance of Accelerated Methods

In this section, we consider the performance of accelerated MP2 and CCSD calculations, whereas accelerated CCSD(T) methods will be assessed in Section 3.2. As detailed above, different statistical measures were collected to quantify the errors. However, all statistical parameters generally provide a similar assessment of the methods. Hence, unless otherwise indicated, we use the relative mean average error (%MAE) to analyze the data.

#### 3.1.1 Accelerated MP2 Calculations

Table 2 reports the statistical measures assessing the performance of six accelerated MP2 methods with respect to canonical MP2. The errors committed by MP2 accelerated methods for the lowest-order properties (dipole moment and polarizability) are minimal (ca. 1%). Therefore, employing any of these methods is advisible to reduce the computational cost of these properties. However, the highest accuracy is achieved by RIJK-MP2, closely followed by RI-MP2 and the analytical calculation of the polarizability at the DLPNO-MP2 level (DLPNO-MP2-α). The remarkable accuracy of the analytical field-free and field-dependent linear polarizabilities obtained from DLPNO-MP2 (used to determine higher-order properties) is reflected by the accuracy of DLPNO-MP2-α first and second hyperpolarizabilities (calculated from numerical derivatives of the former). Together with RI-MP2, DLPNO-MP2-α provides the most accurate values for the whole range of optical properties. Since RIJK-MP2 and RIJCOSX2-MP2 only display a slight increase of the relative MAE on β and γ, while they reduce the computational cost of RI-MP2 by applying the resolution of identity also at the SCF level, one should likewise consider these methods to compute hyperpolarizabilities with values close to the MP2 accuracy.

**Table 2:** Performance of Accelerated MP2 Methods with Respect to Canonical MP2 for the γ-NLO Set^a^

		RI-MP2	RIJK-MP2	RIJCOSX2-MP2	LMP2	DLPNO-MP2-α	DLPNO-MP2
μ	MAE	1.9 × 10^–4^	1.9 × 10^–4^	6.0 × 10^–4^	4.3 × 10^–4^	2.2 × 10^–4^	5.7 × 10^–4^
	RMSE	3.3 × 10^–4^	3.8 × 10^–4^	9.2 × 10^–4^	8.3 × 10^–4^	3.9 × 10^–4^	1.2 × 10^–3^
	MAX	1.2 × 10^–3^	1.4 × 10^–3^	2.6 × 10^–3^	3.1 × 10^–3^	1.5 × 10^–3^	4.7 × 10^–3^
	%MAE	0	0	0	0	0	0
α	MAE	4.6 × 10^–2^	3.9 × 10^–2^	1.2 × 10^0^	3.1 × 10^–1^	7.6 × 10^–2^	9.5 × 10^–1^
	RMSE	1.6 × 10^–1^	1.2 × 10^–1^	7.6 × 10^0^	9.1 × 10^–1^	2.8 × 10^–1^	3.0 × 10^0^
	MAX	1.1 × 10^0^	8.3 × 10^–1^	5.8 × 10^1^	3.9 × 10^0^	1.8 × 10^0^	2.0 × 10^1^
	%MAE	0	0	1	0	0	1
β	MAE	5.7 × 10^–1^	3.7 × 10^0^	1.2 × 10^1^	7.1 × 10^1^	2.9 × 10^0^	1.2 × 10^2^
	RMSE	1.7 × 10^0^	1.5 × 10^1^	3.8 × 10^1^	2.3 × 10^2^	8.9 × 10^0^	2.5 × 10^2^
	MAX	6.6 × 10^0^	7.0 × 10^1^	1.7 × 10^2^	9.9 × 10^2^	3.9 × 10^1^	8.8 × 10^2^
	%MAE	0	2	5	29	1	50
γ	MAE	2.7 × 10^4^	5.5 × 10^4^	5.4 × 10^4^	2.7 × 10^5^	3.3 × 10^4^	1.9 × 10^5^
	RMSE	1.3 × 10^5^	2.6 × 10^5^	1.4 × 10^5^	1.3 × 10^6^	1.5 × 10^5^	4.7 × 10^5^
	MAX	8.7 × 10^5^	1.7 × 10^6^	6.0 × 10^5^	9.5 × 10^6^	1.1 × 10^6^	1.8 × 10^6^
	%MAE	5	11	10	51	6	37

a DLPNO-MP2-α provides analytical values of μ and α, the latter of which is employed to compute the energy derivatives that enter the expressions of β and γ. Units are a.u.

#### 3.1.2 Accelerated CCSD Methods

Table 3 collects data to assess the performance of DLPNO-CCSD and LNO-CCSD against canonical CCSD. The errors committed by CCSD accelerated methods for the dipole moment and the polarizability are slightly larger than their counterparts at the MP2 level, presenting also very small %MAE (ca. equal or below 1%). However, none of these methods provides reasonable values for the first and the second polarizabilities. The excellent results obtained from analytical DLPNO-MP2-α suggest that if analytical values of the polarizability at the DLPNO-CCSD were available, we would also obtain accurate hyperpolarizabilities at this level of theory.

**Table 3:** Performance of Accelerated CCSD Methods with Respect to Canonical CCSD for the γ-NLO Set^a^

		DLPNO-CCSD	LNO-CCSD
μ	MAE	2.4 × 10^–3^	1.6 × 10^–3^
	RMSE	4.7 × 10^–3^	3.5 × 10^–3^
	MAX	1.8 × 10^–2^	1.3 × 10^–2^
	%MAE	0	0
α	MAE	1.4 × 10^0^	1.6 × 10^0^
	RMSE	5.2 × 10^0^	7.1 × 10^0^
	MAX	3.7 × 10^1^	4.4 × 10^1^
	%MAE	1	1
β	MAE	1.1 × 10^2^	5.1 × 10^1^
	RMSE	2.3 × 10^2^	1.2 × 10^2^
	MAX	6.5 × 10^2^	4.6 × 10^2^
	%MAE	62	28
γ	MAE	3.7 × 10^5^	1.1 × 10^5^
	RMSD	9.0 × 10^5^	4.1 × 10^5^
	MAX	5.9 × 10^6^	2.6 × 10^6^
	%MAE	97	29

a Units are a.u.

The poor results obtained for the static high-order optical properties from DLPNO-CCSD and LNO-CCSD are due to numerical errors (see Table S10 and Table S11 for the Romberg errors) that could not be avoided using other numerical differentiation techniques. The poor precision of the single-point DLPNO-CCSD and LNO-CCSD field-dependent energies is responsible for it, and it cannot be solved by using tighter convergence criteria for the localization schemes. Table S12 shows that these results are even worse if we employ looser localization criteria.

### 3.2 Absolute Performance of Accelerated Methods

Thus, far, we have evaluated the efficiency of accelerated MP2 and CCSD to reproduce their canonical counterparts. In this section, we benchmark accelerated methods against the reference CCSD(T) calculations for the calculation of SNLOPs, including accelerated CCSD(T) variants, which are assessed for the first time.

Table 4 collects the statistical data for the dipole moment and the polarizability. Accelerated MP2 and CCSD methods are omitted from these analyses, since we demonstrated above that they have similar accuracy to their canonical counterparts (see Table 2). DLPNO-CCSD(T) variants show excellent performance with errors below or equal to 1% with respect to the canonical CCSD(T). MP2 and CCSD methods also provide very good approximations of the two properties.

**Table 4:** Performance of acceleration methods with respect to CCSD(T) references for the evaluation of μ and α for the γ-NLO set. Units are a.u

		DLPNO-CCSD(T0)	DLPNO-CCSD(T1)	LNO-CCSD(T)	MP2	CCSD
μ	MAE	3.4 × 10^–3^	2.0× 10^–3^	2.3 × 10^–3^	2.9 × 10^–2^	2.2 × 10^–2^
	RMSE	6.6 × 10^–3^	5.1 × 10^–3^	6.3 × 10^–3^	4.3 × 10^–2^	3.4 × 10^–2^
	MAX	2.5 × 10^–2^	2.0 × 10^–2^	2.6 × 10^–2^	1.3 × 10^–1^	9.5 × 10^–2^
	%MAE	0	0	0	4	3
α	MAE	1.5 × 10^0^	8.2 × 10^–1^	1.2 × 10^0^	3.7 × 10^0^	3.0 × 10^0^
	RMSE	4.2 × 10^0^	2.1 × 10^0^	3.9 × 10^0^	7.3 × 10^0^	8.8 × 10^0^
	MAX	2.0 × 10^1^	1.0 × 10^1^	2.1 × 10^1^	3.1 × 10^1^	5.9 × 10^1^
	%MAE	1	1	1	3	3

The data in Tables 5 and 6 illustrate the performance of various methods to compute the first and second hyperpolarizability, respectively. None of the methods tested give a relative MAE below 15% for the first hyperpolarizability, the best methods being DLPNO-CCSD(T1) followed by canonical CCSD. The latter results indicate that the accurate evaluation of triples is crucial in reproducing CCSD(T) values. All DLPNO-based methods show significant numerical derivative errors (see MARoE values in Table S10 and Table S11), which can be partially (but not sufficiently) reduced by employing tighter cutoffs (see Table S13). Interestingly, the ability of DLPNO methods in reproducing triple excitations goes hand in hand with the numerical stability of the energies. Indeed, for the same cutoffs, we find more precise energies (lower MARoE values) for DLPNO-CCSD(T1) than for DLPNO-CCSD(T0)—see Table S13. The LNO-CCSD method gives somewhat more accurate first hyperpolarizabilities than DLPNO-CCSD. Conversely, DLPNO-CCSD(T0) is more accurate than LNO-CCSD(T). However, both LNO methods exhibit relative MAE errors above 60% and should not be considered for the computation of β. Interestingly, LMP2 outperforms MP2, mainly because of a better reproduction of the first hyperpolarizabilities of the PMI oligomers by LMP2—which is probably due to a fortuitous cancellation of errors.

**Table 5:** Performance of Acceleration Methods with Respect to CCSD(T) References for the Evaluation of β for the γ-NLO Set

		DLPNO			LNO			Canonical	
		CCSD	CCSD(T0)	CCSD(T1)	CCSD	CCSD(T)	LMP2	MP2	CCSD
β	MAE	1.3 × 10^2^	8.9 × 10^1^	2.5 × 10^1^	9.1 × 10^1^	9.2 × 10^1^	5.7 × 10^1^	1.1 × 10^2^	5.1 × 10^1^
	RMSE	2.9 × 10^2^	2.9 × 10^2^	4.8 × 10^1^	2.5 × 10^2^	3.4 × 10^2^	1.2 × 10^2^	2.6 × 10^2^	1.3 × 10^2^
	MAX	1.1 × 10^3^	1.3 × 10^3^	1.9 × 10^2^	9.2 × 10^2^	1.6 × 10^3^	3.5 × 10^2^	8.3 × 10^2^	4.6 × 10^2^
	%MAE	91	65	18	66	67	41	78	37

**Table 6:** Performance of Acceleration Methods with Respect to CCSD(T) References for the Evaluation of γ for the γ-NLO Set^a^

		DLPNO			LNO			Canonical	
		CCSD	CCSD(T0)	CCSD(T1)	LNO-CCSD	LNO-CCSD(T)	LMP2	MP2	CCSD
γ	MAE	4.3 × 10^5^	1.8 × 10^5^	1.5 × 10^5^	1.5 × 10^5^	1.8 × 10^5^	2.6 × 10^5^	7.7 × 10^4^	8.4 × 10^4^
	RMSE	9.7 × 10^5^	7.0 × 10^5^	5.3 × 10^5^	7.2 × 10^5^	1.0 × 10^6^	1.2 × 10^6^	2.4 × 10^5^	3.7 × 10^5^
	MAX	5.9 × 10^6^	5.1 × 10^6^	3.3 × 10^6^	5.3 × 10^6^	7.7 × 10^6^	8.9 × 10^6^	1.2 × 10^6^	2.7 × 10^6^
	%MAE	94	39	33	32	41	58	17	19

a Units are a.u.

In Table 6, we collect the statistics for the second hyperpolarizabilities. In this case, none of the accelerated methods gives a relative MAE below 30%, the lowest error being for DLPNO-CCSD(T1) (we chose this method over LNO-CCSD because the latter presents larger RMSE and MAX errors). All the methods also show substantial numerical derivative errors, evidencing that numerical instabilities in the energy values hinder an accurate calculation of their fourth-order derivatives (see Tables S9 and S10). Interestingly, MP2 performs better than CCSD. In this sense, RI-based accelerated MP2 methods (see Table 2) are an economical alternative for computing second hyperpolarizabilities.

### 3.3 Performance of Spin-Component Scaled Methods

In this section, we assess the accuracy of several spin-component scaled methods designed to improve the performance of MP2 energies by adjusting the amount of same-spin (*c*_SS_) and opposite-spin (*c*_OS_) correlations. As described above, we tested two different popular schemes: the Grimme’s SCS-MP2^85^ in which *c*_SS_ = 1/3 and *c*_OS_ = 6/5 and SOS-MP2^103^ that takes *c*_SS_ = 0 and *c*_OS_ = 1.3. The computational cost of SCS-MP2 is identical to canonical MP2, whereas SOS-MP2 can be formally implemented to reduce the scaling by one order in the number of basis functions. We collected the statistics of their performance in Table 7.

**Table 7:** Performance of Different Spin-Component Scaled MP2 Methods with Respect to CCSD(T) for the Evaluation of the Linear and Nonlinear Optical Properties for the γ-NLO Set^a^

		MP2	SCS-MP2	SOS-MP2
	*c*_*SS*_	1	1/3	0
	*c*_*OS*_	1	6/5	1.3
μ	MAE	2.9 × 10^–2^	2.0 × 10^–2^	1.5 × 10^–2^
	RMSE	4.3 × 10^–2^	2.7 × 10^–2^	1.9 × 10^–2^
	MAX	1.3 × 10^–1^	6.6 × 10^–2^	4.3 × 10^–2^
	%MAE	4	3	2
α	MAE	3.7 × 10^0^	2.9 × 10^0^	4.8 × 10^0^
	RMSE	7.2 × 10^0^	8.4 × 10^0^	1.4 × 10^1^
	MAX	3.1 × 10^1^	5.0 × 10^1^	8.3 × 10^1^
	%MAE	3	3	4
β	MAE	1.1 × 10^2^	1.7 × 10^2^	2.0 × 10^2^
	RMSE	2.6 × 10^2^	4.4 × 10^2^	5.4 × 10^2^
	MAX	8.3 × 10^2^	1.6 × 10^3^	2.0 × 10^3^
	%MAE	78	125	148
γ	MAE	7.7 × 10^4^	6.4 × 10^4^	5.6 × 10^4^
	RMSE	2.4 × 10^5^	1.9 × 10^5^	1.7 × 10^5^
	MAX	1.2 × 10^6^	9.3 × 10^5^	8.5 × 10^5^
	%MAE	17	14	12

a Units are a.u.

The results for the dipole moment and the polarizability show only a marginal improvement over MP2, which already exhibits pretty accurate results. According to all statistical measures, the first hyperpolarizability is better estimated by canonical MP2 than by spin-scaled methods. Reversely, both SCS-MP2 and SOS-MP2 marginally better reproduce the second hyperpolarizability. Although there is not much improvement, it might be worth exploring the possibility of an accelerated SOS-MP2 method as an economical way to compute γ.

### 3.4 Performance of Accelerated Methods on Push–Pull Systems

From the results of the previous sections, we have identified β and γ as the most challenging properties for accelerated wave function methods. We have also identified DLPNO-CCSD(T1) as the best-performing method for β, whereas for γ the only accelerated wave function methods we can use are the RI variants of MP2. In this section, we put these methods to the test by analyzing further their relative accuracy for computing the NLO properties of the push–pull molecules contained in the β-NLO set. These molecules are expected to exhibit a significant β response that is susceptible to be impacted by larger errors. The statistical results are collected in Table 8.

**Table 8:** Performance of DLPNO-CCSD(T1), MP2, RI-MP2, and CCSD Methods with Respect to the CCSD(T) References for the Evaluation of β and γ for the β-Set^a^

		DLPNO-CCSD(T1)	RI-MP2	MP2	CCSD
β	MAE	1.5 × 10^3^	2.8 × 10^3^	2.8 × 10^3^	7.7 × 10^2^
	RMSE	2.4 × 10^3^	4.5 × 10^3^	4.5 × 10^3^	1.2 × 10^3^
	MAX	6.8 × 10^3^	1.3 × 10^4^	1.3 × 10^4^	4.5 × 10^3^
	%MAE	15	28	28	8
γ	MAE	1.6 × 10^6^	5.6 × 10^5^	5.2 × 10^5^	3.9 × 10^5^
	RMSE	3.7 × 10^6^	9.8 × 10^5^	8.7 × 10^5^	7.0 × 10^5^
	MAX	1.7 × 10^7^	3.1 × 10^6^	2.9 × 10^6^	3.2 × 10^6^
	%MAE	60	21	19	14

a Units are a.u.

Although the molecules in the β-NLO set present larger absolute errors than those in the γ-NLO set, the relative errors are smaller because of the larger average β and γ values. The performance of DLPNO-CCSD(T1) compared to CCSD is not as good as in the γ-NLO set. DLPNO-CCSD(T1) first hyperpolarizabilities have average errors about twice as large as those of CCSD. For γ, DLPNO-CCSD(T1) is highly affected by the numerical errors and its performance deteriorates (%MAE = 60%). The results of MP2 and RI-MP2 are comparable for the first and second polarizabilities, showing that the resolution of identity methods can be safely employed as substitutes for MP2 also for π-conjugated push–pull derivatives. The comparison between MP2 and CCSD shows that, for molecules with larger responses, MP2 exhibits larger deviations than CCSD for the first hyperpolarizability, while both methods lead to similar %MAEs for the second hyperpolarizability.

## 4 Conclusions

In this paper, we have benchmarked various alternatives to wave function methods that either reduce the computational cost or improve the performance of the canonical methods. In particular, we have tested RI-MP2, RIJK-MP2, RIJCOSX2-MP2, LMP2, SCS-MP2, SOS-MP2, DLPNO-MP2, LNO-CCSD, LNO-CCSD(T), DLPNO-CCSD, DLPNO-CCSD(T0), and DLPNO-CCSD(T1). Our results indicate that all these methods produce numerically stable energies to compute their first and second derivatives with respect to an external electric field. Since, in general, these derivatives are not highly affected by correlation energy, we can safely employ any of the latter methods to calculate the dipole moment and the polarizability with average relative errors below 5%.

On the other hand, the calculation of higher-order derivatives represents a challenge for both accelerated and enhanced wave function methods. In particular, the third and fourth derivatives of the energy (required to compute the first and second polarizabilities) critically depend on the numerical stability of the single-point field-dependent energy calculations.

Our results show that RI-based methods produce reliable energies from which to compute up to fourth-order derivatives of the energy with respect to an external field. Hence, methods like RI-MP2, RIJK-MP2, or RIJCOSX2-MP2 are a cost-effective way to obtain first and second hyperpolarizabilities with a marginal average error with respect to canonical MP2 (up to 5% for β and up to 11% for γ). Conversely, methods based on orbital localizations (LNO and DLPNO techniques) applied to MP2 suffer from large numerical instabilities that result in large errors for β (29–50%) and γ (37–51%). The same techniques applied to CCSD and CCSD(T) result in even larger errors, which are close to 100% in the worse cases. The only exception is DLPNO-CCSD(T1), which produces an acceptable relative error of 18% for the calculation of β with *tight* cutoffs. Due to the numerical stability of the single-point energies, among the methods tested, the most accurate results for γ are obtained with MP2-based methods.

The precision of single-point energy calculations with LNO and DLPNO critically depends on the cutoffs for the SCF/CC equations and the orbital localization scheme; in particular, tight localization criteria for the construction of LNO or DLPNO are essential. In addition, the ability of DLPNO methods in reproducing triple excitations goes hand in hand with the numerical stability of the energies. Hence, for the same cutoffs, we have more precise energies and, consequently, more accurate electric properties for DLPNO-CCSD(T1) than for DLPNO-CCSD(T0).

Analytical field-dependent polarizabilities are available at the DLPNO-MP2 level of theory, from which we have numerically computed first and second hyperpolarizabilities that are in excellent agreement with their canonical MP2 counterparts. Since canonical CCSD is often the most accurate method after CCSD(T), we can anticipate that, if analytical DLPNO-CCSD polarizabilities were available, we would have a cost-effective method to compute accurate first and second hyperpolarizabilities.

Finally, we assessed spin-component scaled methods as techniques for improving the performance of MP2 at the same cost. However, these techniques produce only a marginal improvement in the case of the second polarizability.

Based on a computational cost assessment (see Section 1 of the Supporting Information), we recommend RIJK-MP2 and RIJCOSX2-MP2 to compute static dipole moments, polarizabilities, and second hyperpolarizabilities, whereas only DLPNO-CCSD(T1) using tight cutoffs can be employed to obtain reasonably accurate static first hyperpolarizabilities. Although DLPNO-MP2 also provides excellent results (using analytical polarizabilities to compute γ), its computational cost is clearly above the latter RI methods, coming very close to the canonical MP2 method (see Table S4). On the other hand, the computational gain of DLPNO-CCSD methods is enormous compared to their canonical counterparts (see Figure S2). We hope that these results will prompt the implementation of analytical low-order properties for accelerated wave function methods and/or more *precise* single-point energies that can be employed to compute numerical derivatives.

## Supporting Information Available

Data of linear and nonlinear optical property calculations (PDF)
Summary data (XLSX)
