# Supplementary material for: Are Accelerated and Enhanced Wave Function Methods Accurate to Compute Static Linear and Nonlinear Optical Properties?
Source: J Chem Theory Comput. 2023 Mar 2;19(6):1753–64. doi: 10.1021/acs.jctc.2c01212 (PMC10061658; doi:10.1021/acs.jctc.2c01212)
Supplement: Supplementary file 1 — ct2c01212_si_002.pdf [file ct2c01212_si_002.pdf]

Supporting Information: Are accelerated and enhanced wavefunction methods accurate to compute static linear and nonlinear optical properties?

Carmelo Naim,[†,‡,¶,§] Pau Besalú-Sala,[‖,§] Robert Zaleśny,[⊥] Josep M. Luis,[*,‖] Frédéric Castet,[*,‡] and Eduard Matito[*,†,#]

†*Donostia International Physics Center (DIPC), Manuel Lardizabal Ibilbidea 4, 20018 Donostia, Euskadi, Spain*

‡*Univ. Bordeaux, CNRS, Bordeaux INP, ISM, UMR 5255, F-33400 Talence, France*

¶*Polimero eta Material Aurreratuak: Fisika, Kimika eta Teknologia, Kimika Fakultatea, Euskal Herriko Unibertsitatea UPV/ EHU, 20080 Donostia, Euskadi, Spain*

§*These authors have equally contributed to this work*

‖*Institut de Química Computacional i Catàlisi and Departament de Química, Universitat de Girona, 17003 Girona, Catalonia, Spain*

⊥*Faculty of Chemistry, Wrocław University of Science and Technology, Wyb. Wyspiańskiego 27, PL−50370 Wrocław, Poland*

#*Ikerbasque Foundation for Science, 48011 Bilbao, Euskadi, Spain*

E-mail: josepm.luis@udg.edu; frederic.castet@u-bordeaux.fr; ematito@gmail.com

Contents

1 Computational time **S-2**

1.0.1 Comparison between DLPNO-MP2 and DLPNO-MP2-α S-8

2 Indicators of correlation **S-10**

3 Numerical errors **S-14**

4 Impact of the different convergence criteria on the accuracy of the localization schemes **S-16**

5 Performances of accelerated methods on the β-Set **S-21**

6 Performances of the accelereted methods merging the β-NLO and γ-NLO-B sets **S-22**

7 Cartesian coordinates for the molecules of the γ-NLO and β-NLO sets **S-22**

1 Computational time

Before computing the optical properties of the molecules belonging to the γ-NLO set, we performed a test to have a qualitative idea about the relative computational time required for computing the electrical (hyper)polarizabilities with all the methods considered in this study. Regarding CCSD(T) and CCSD calculations, we performed field-free single-point (SP) calculations on the five PA oligomers of the γ-NLO set, as they cover a reasonable range of molecular sizes representative of the whole set. For MP2, the SP calculations were much faster. For this reason, we decided to switch from PA oligomers to PDA oligomers, which are the biggest molecules of the γ-NLO set. All the calculations have been performed on a single node with 16 cpus Intel Xeon Gold 6140, with a disk memory of 384 GB and 64 GB of RAM, with the only exception of MP2 calculations, which were computed on a single

cpu. The wall time (in minutes) of each calculation is collected in Tables S1 to S3. The computational scaling of these methods with respect to the number of basis functions has been evaluated by a polynomial fit. The fitting has been performed by adjusting a and b in the function $y = ax^b$. We also included CAM-B3LYP SP calculations for the same molecules in order to verify how these methods perform time-wise with respect to a range separated density functional approach time scaling. The results are collected in Figures S1 to S3 and in Table S4.

From the resulting fit of CC approximations, in general CCSD and CCSD(T) are faster than their corresponding RI counterparts. This behavior is associated to the density fitting implementations, which might not be optimal for these methods. On the contrary, when employing RI-MP2 and RIJK-MP2 schemes, the computational cost drops substantially with respect to MP2. Regarding the RIJCOSX method, we observed that moving from the default COSX grid to the COSX2 one (which is a more dense grid) does not substantially affect the cost, and therefore only the second one is presented in the manuscript.

Regarding the localized methods, moving to tighter convergence criteria does not drastically increase the computational cost, and therefore, owing to the huge accuracy gained in the NLOPs, we only present the calculations employing the Tight criteria on the DLPNO and the veryTight criteria on the LNO construction. For CCSD and CCSD(T) calculations, LNO based methods generally require more time than the corresponding DLPNO counterparts. On the other hand, T1 corrections for DLPNO-CCSD present a smaller exponent with respect to standard T corrections, but the parameter a is one order of magnitude larger, indicating that time savings start to become relevant only for large molecules (more than 400 basis functions). To finalize, DLPNO-MP2 schemes show comparable timings with respect to MP2. Instead, LMP2 is more efficient than DLPNO and shows a comparable scaling to CAM-B3LYP.

Table S1: Wall time for approximate CCSD calculations tested on PA chains of different lengths. These calculations have been performed employing 16 cpus. The values in the table are in minutes per cpu.

			DLPNO-CCSD		LNO-CCSD		
**Molecule**	**CCSD**	**RI-CCSD**	**Normal**	**Tight**	**Normal**	**Tight**	**VeryTight**
PA1	3	25	12	5	5	5	5
PA2	14	53	7	14	19	19	24
PA3	52	260	15	31	46	59	87
PA4	185	1041	28	67	96	140	234
PA5	594	3349	44	95	175	259	469
PA6	1538	8803	71	139	281	423	773

Table S2: Wall time for approximate CCSD(T) calculations tested on PA chains of different lengths. These calculations have been performed employing 16 cpus. The values in the table are in minutes per cpu.

			DLPNO-CCSD(T0)		DLPNO-CCSD(T1)		LNO-CCSD(T)		
**Molecule**	**CCSD(T)**	**RI-CCSD(T)**	**Normal**	**Tight**	**Normal**	**Tight**	**Normal**	**Tight**	**vTight**
PA1	18	6	4	6	12	6	5	7	6
PA2	12	64	9	15	23	31	20	27	36
PA3	78	253	18	34	38	39	63	86	135
PA4	301	1083	34	78	73	83	131	203	350
PA5	1068	3572	50	113	111	121	221	374	737
PA6	3212	9519	73	172	146	171	339	576	1171

Table S3: Wall time for approximate MP2 calculations tested on PDA chains of different lengths. These calculations have been performed employing 1 cpu. The values in the table are in minutes per cpu.

			RI-MP2			DLPNO-MP2		LMP2		
**Molecule**	**MP2**	**SCF**	**RI-JK-SCF**	**RI-JCOSX**	**RI-JCOSX2**	**Normal**	**Tight**	**Normal**	**Tight**	**vTight**
PDA1	3	2	1	1	2	5	3	1	1	1
PDA2	9	7	2	2	4	11	12	5	5	5
PDA3	22	16	4	5	8	28	27	12	12	12
PDA4	46	31	8	8	13	48	50	23	23	27
PDA5	72	48	14	13	21	72	84	39	37	45

Figure S1: Wall time for approximate CCSD calculations tested on PA chains of different lengths with respect to the number of basis functions, and the corresponding best fits. These calculations have been performed employing 16 cpus. The time values are in minutes per cpu.

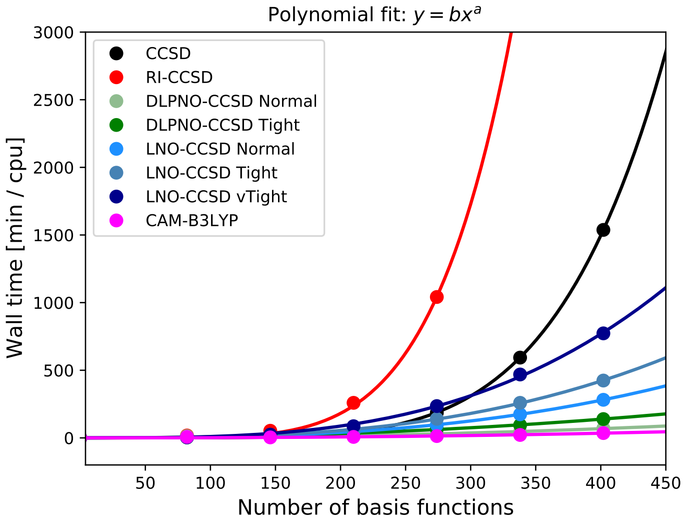

Figure S2: Wall time (in minutes) for approximate CCSD(T) calculations tested on PA chains of different lengths with respect to the number of basis functions, and the corresponding best fits. Data computed using 16 CPUs in a single node.

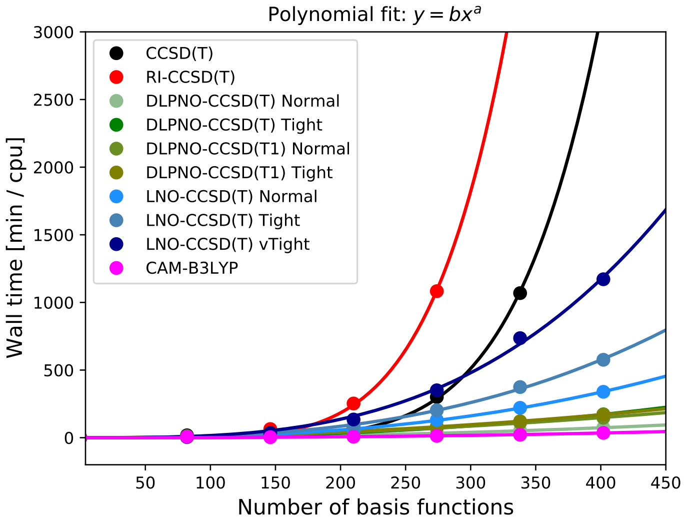

Figure S3: MP2 timings (in minutes per cpu) for the PDA oligomers with respect to the number of basis functions. Data computed using one CPU.

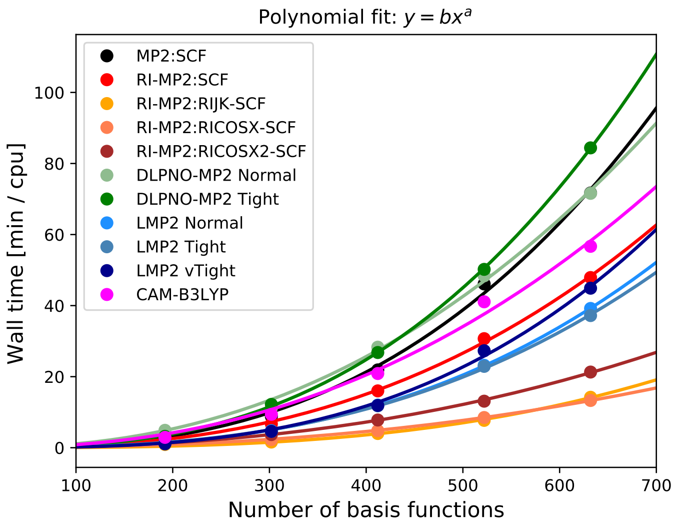

Table S4: Parameters obtained from the best fits of the wall time with respect to the number of basis functions of approximate CCSD(T), CCSD, and CAM-B3LYP calculations, performed using 16 cpus for the PA chains, and from those of MP2 and CAM-B3LYP calculations performed with one cpu for the PDA chains.

**Method**	**Approx**	a	b **[min / cpu]**	r^2
CCSD(T)	-	6.28	2.30E-12	1.00
RI-CCSD(T)	-	5.65	2.88E-10	1.00
DLPNO-CCSD(T0)	Normal	2.13	3.73E-03	1.00
DLPNO-CCSD(T0)	Tight	2.34	2.23E-03	1.00
DLPNO-CCSD(T1)	Normal	1.90	2.69E-02	1.00
DLPNO-CCSD(T1)	Tight	1.97	2.04E-02	1.00
LNO-CCSD(T)	Normal	2.56	1.20E-03	1.00
LNO-CCSD(T)	Tight	2.76	5.88E-04	1.00
LNO-CCSD(T)	vTight	3.10	1.62E-04	1.00
CCSD	-	5.48	8.24E-12	1.00
RI-CCSD	-	5.56	2.87E-11	1.00
DLPNO-CCSD	Normal	2.17	1.57E-04	0.97
DLPNO-CCSD	Tight	2.13	4.05E-04	1.00
LNO-CCSD	Normal	2.77	1.72E-05	1.00
LNO-CCSD	Tight	2.93	1.02E-05	1.00
LNO-CCSD	vTight	3.13	5.36E-06	1.00
MP2:SCF	-	2.68	1.33E-04	1.00
RI-MP2:SCF	-	2.55	2.13E-04	1.00
RI-MP2:RIJK-SCF	-	2.98	3.74E-06	1.00
RI-MP2:RICOSX-SCF	-	2.33	2.43E-04	1.00
RI-MP2:RICOSX2-SCF	-	2.35	3.33E-04	1.00
DLPNO-MP2	Normal	2.27	1.92E-03	1.00
DLPNO-MP2	Tight	2.68	1.62E-04	1.00
LMP2	Normal	2.78	3.88E-05	1.00
LMP2	Tight	2.70	5.99E-05	1.00
LMP2	vTight	2.96	1.43E-05	1.00
CAM-B3LYP (from PA)	-	2.35	4.20E-04	0.95
CAM-B3LYP (from PDA)	-	2.28	1.40E-03	0.99

1.0.1 Comparison between DLPNO-MP2 and DLPNO-MP2-α

We compare here the performance of analytic DLPNO-MP2 calculations for computing polarizabilities (DLPNO-MP2-α) with the values obtained from numerical energy derivatives at the DLPNO-MP2 level using Orca 5.

Table S5: Wall time for approximate DLPNO-MP2 calculations tested on the PDA chains of different lengths. These calculations have been performed employing 16 cpus. The values in the table are in minutes per cpu. DLPNO-MP2 timings have been multiplied by 9 (the number of field-dependent single-point calculations required to compute α with our numerical procedure) for comparison.

	DLPNO-MP2		DLPNO-MP2-α	
**Molecule**	**Normal**	**Tight**	**Normal**	**Tight**
PDA1	86	86	32	48
PDA2	158	187	144	144
PDA3	331	389	272	304
PDA4	576	648	448	528
PDA5	864	1022	704	848

Figure S4: Wall time (in minutes per cpu) for approximate DLPNO-MP2 calculations tested on the PDA chains of different lengths with respect to the number of basis functions and the corresponding best fit. Data computed using 16 CPUs in a single node.

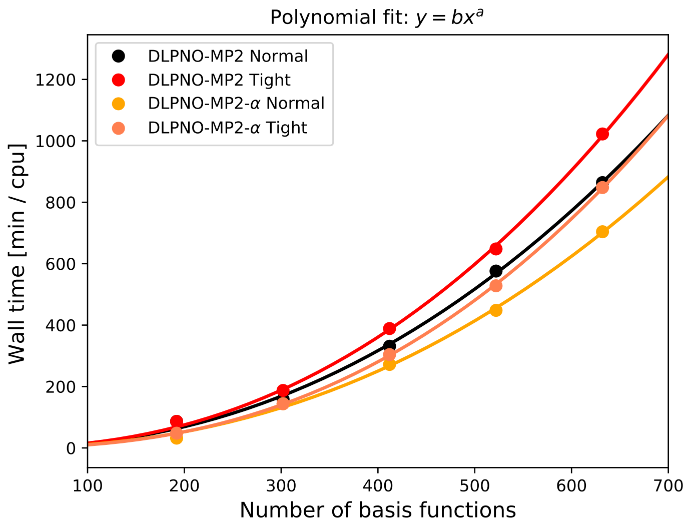

Table S6: Parameters obtained from the best fits of the wall time with respect to the number of basis functions of approximate MP2 calculations computed with 16 cpus for the PDA chains.

**Method**	**Approx**	a	b **[min / cpu]**	r^2
DLPNO-MP2	Normal	2.20	6.07E-04	1.00
DLPNO-MP2	Tight	2.26	4.62E-04	1.00
DLPNO-MP2-α	Normal	2.25	3.43E-04	1.00
DLPNO-MP2-α	Tight	2.41	1.48E-04	1.00

2 Indicators of correlation

Table S7: D1, D2, and T1 diagnostics calculated on the CCSD(T) wavefunction for each molecule of the γ-NLO-A and γ-NLO-B sets. In red are highlighted the molecules which show multireference character according to the indicator proposed, namely: $T_1 \geq 0.02$, $D_1 \geq 0.05$ and $D_2 \geq 0.18$.

Molecule	D1	D2	T1	Molecule	D1	D2	T1
Carbon monoxide	0.042	0.164	0.020	Pentane	0.017	0.146	0.008
Hydrogen fluoride	0.022	0.120	0.013	Cyclohexane	0.018	0.146	0.009
Dinitrogen	0.024	0.175	0.013	Hexane	0.017	0.147	0.008
Dioxygen	0.038	0.245	0.016	Heptane	0.017	0.147	0.008
Water	0.027	0.127	0.012	Octane	0.018	0.148	0.008
Carbon dioxide	0.051	0.156	0.020	H_2	0.017	0.184	0.012
Cyanogen fluoride	0.031	0.180	0.016	$(H_2)_2$	0.017	0.191	0.011
Hydrogen cyanide	0.028	0.185	0.015	$(H_2)_3$	0.017	0.193	0.011
Hydrogen isocyanide	0.028	0.185	0.015	$(H_2)_4$	0.017	0.195	0.011
Nitroxyl	0.045	0.213	0.018	$(H_2)_5$	0.017	0.195	0.011
Dinitrogen oxide	0.049	0.192	0.021	$(H_2)_6$	0.017	0.196	0.011
Ammonia	0.023	0.134	0.010	$(H_2)_7$	0.017	0.196	0.011
Acetylene	0.027	0.188	0.013	$(H_2)_8$	0.017	0.197	0.011
Formaldehyde	0.048	0.186	0.017	PMI1	0.034	0.197	0.013
Hydrogen peroxide	0.030	0.183	0.015	PMI2	0.046	0.208	0.015
Nitrous acid	0.067	0.199	0.024	PMI3	0.058	0.213	0.016
Nitric acid	0.065	0.200	0.020	PMI4	0.070	0.215	0.017
Methane	0.011	0.135	0.007	PMI5	0.081	0.216	0.018
Carbonic acid	0.058	0.158	0.016	PMI6	0.090	0.217	0.019
Boric acid	0.048	0.129	0.014	PA1	0.028	0.197	0.010
Ethane	0.014	0.140	0.007	PA2	0.031	0.211	0.011
Propane	0.015	0.143	0.008	PA3	0.033	0.218	0.011
1-butene	0.029	0.197	0.010	PA4	0.035	0.222	0.011
Benzene	0.024	0.191	0.010	PA5	0.036	0.225	0.011
Butane	0.016	0.144	0.008	PA6	0.037	0.227	0.011
1-butanol	0.029	0.145	0.010	PDA1	0.033	0.208	0.011
Pentanal	0.051	0.179	0.013	PDA2	0.033	0.213	0.012
n-butylamine	0.027	0.145	0.009	PDA3	0.033	0.215	0.012
1-pentanoic acid	0.058	0.163	0.014	PDA4	0.034	0.220	0.012
1-pentanamide	0.059	0.163	0.013	PDA5	0.036	0.232	0.012

Table S8: Indicators based on natural orbital occupancies on the MP2 wavefunction for each molecule of the γ-NLO-A and γ-NLO-B sets. In red are highlighted the molecules which have a multireference character according to the indicator proposed. The threshold values have been published for a CCSD wavefunction: $NON \geq 0.058$, $\bar{V} \geq 0.03$, $MRI \leq 0$, and $I_{ND} \geq 0.03$.

**Molecule**	NON	$\bar{V}$	MRI	I_{ND}
Carbon monoxide	0.026	0.025	0.981	0.014
Hydrogen fluoride	0.012	0.017	0.998	0.009
Dinitrogen	0.032	0.029	0.935	0.016
Dioxygen	0.036	0.024	0.935	0.013
Water	0.013	0.020	0.997	0.012
Carbon dioxide	0.031	0.024	0.967	0.013
Cyanogen fluoride	0.033	0.025	0.926	0.014
Hydrogen cyanide	0.032	0.030	0.919	0.016
Hydrogen isocyanide	0.032	0.030	0.919	0.016
Nitroxyl	0.038	0.026	0.916	0.014
Dinitrogen oxide	0.048	0.031	0.603	0.017
Ammonia	0.011	0.021	0.997	0.012
Acetylene	0.027	0.028	0.943	0.016
Formaldehyde	0.029	0.023	0.977	0.013
Hydrogen peroxide	0.030	0.023	0.971	0.013
Nitrous acid	0.034	0.024	0.941	0.013
Nitric acid	0.046	0.025	0.854	0.014
Methane	0.008	0.018	0.998	0.011
Carbonic acid	0.027	0.021	0.983	0.012
Boric acid	0.018	0.020	0.991	0.011
Ethane	0.011	0.019	0.995	0.012
Propane	0.012	0.020	0.992	0.012
1-butene	0.024	0.022	0.971	0.013
Benzene	0.035	0.026	0.786	0.015
Butane	0.013	0.020	0.990	0.012
1-butanol	0.017	0.021	0.987	0.012
Pentanal	0.028	0.021	0.971	0.012
n-butylamine	0.015	0.021	0.987	0.012
1-pentanoic acid	0.027	0.021	0.974	0.012
1-pentanamide	0.027	0.021	0.974	0.012

**Molecule**	NON	$\bar{V}$	MRI	I_{ND}
Pentane	0.013	0.020	0.987	0.012
Cyclohexane	0.013	0.021	0.983	0.012
Hexane	0.014	0.020	0.985	0.012
Heptane	0.014	0.021	0.982	0.012
Octane	0.015	0.021	0.980	0.012
H_2	0.009	0.023	0.999	0.014
$(H_2)_2$	0.012	0.025	0.999	0.015
$(H_2)_3$	0.013	0.025	0.998	0.015
$(H_2)_4$	0.014	0.026	0.997	0.016
$(H_2)_5$	0.014	0.026	0.996	0.016
$(H_2)_6$	0.014	0.026	0.996	0.016
$(H_2)_7$	0.015	0.026	0.995	0.016
$(H_2)_8$	0.015	0.026	0.994	0.016
PMI1	0.028	0.024	0.972	0.014
PMI2	0.032	0.025	0.931	0.014
PMI3	0.034	0.025	0.892	0.014
PMI4	0.034	0.025	0.865	0.014
PMI5	0.034	0.025	0.847	0.014
PMI6	0.034	0.025	0.835	0.014
PA2	0.022	0.022	0.982	0.013
PA4	0.029	0.023	0.947	0.014
PA6	0.033	0.024	0.902	0.014
PA8	0.036	0.024	0.853	0.014
PA10	0.037	0.024	0.806	0.014
PA12	0.039	0.024	0.763	0.014
PDA1	0.035	0.025	0.887	0.014
PDA2	0.039	0.026	0.784	0.015
PDA3	0.041	0.026	0.695	0.015
PDA4	0.043	0.026	0.618	0.015
PDA5	0.043	0.027	0.550	0.015

Table S9: T1 diagnostic calculated on the CCSD wavefunction for each molecule of the β-NLO set. In red are highlighted the molecules which show multireference character: $T_1 \geq 0.02$

Molecule	T1	Molecule	T1
PA2-NH2-CHO	0.017	PMI4-OH-NO2	0.021
PA2-NH2-NO2	0.020	PMI5-NH2-CHO	0.020
PA2-OH-CHO	0.017	PMI5-OH-CHO	0.020
PA2-OH-NO2	0.020	PMI5-OH-NO2	0.022
PA4-NH2-CHO	0.016	PA10-NH2-CHO	0.015
PA4-NH2-NO2	0.020	PA10-NH2-NO2	0.018
PA4-OH-CHO	0.016	PA10-OH-CHO	0.015
PA4-OH-NO2	0.019	PA10-OH-NO2	0.018
PA6-NH2-CHO	0.016	PA12-NH2-CHO	0.015
PA6-OH-CHO	0.016	PA12-NH2-NO2	0.017
PDA1-CHO-OH	0.017	PA12-OH-CHO	0.015
PDA1-NH2-CHO	0.016	PA12-OH-NO2	0.017
PDA1-OH-NO2	0.019	PA6-OH-NO2	0.019
PMI2-NH2-CHO	0.019	PA8-NH2-CHO	0.016
PMI2-NH2-NO2	0.021	PA8-NH2-NO2	0.018
PMI2-OH-CHO	0.019	PDA2-NH2-CHO	0.015
PMI2-OH-NO2	0.021	PDA2-NH2-NO2	0.018
PMI3-NH2-CHO	0.019	PDA2-OH-CHO	0.015
PMI3-NH2-NO2	0.021	PDA2-OH-NO2	0.018
PMI3-OH-CHO	0.019	PDA3-NH2-CHO	0.015
PMI3-OH-NO2	0.021	PDA3-NH2-NO2	0.017
PMI4-OH-CHO	0.019	PDA3-OH-CHO	0.015
PA6-NH2-NO2	0.019	PDA3-OH-NO2	0.017
PA8-OH-CHO	0.015	PMI5-NH2-NO2	0.022
PA8-OH-NO2	0.018	PMI6-NH2-CHO	0.020
PDA1-NH2-NO2	0.019	PMI6-NH2-NO2	0.022
PMI4-NH2-CHO	0.020	PMI6-OH-CHO	0.020
PMI4-NH2-NO2	0.021	PMI6-OH-NO2	0.022

3 Numerical errors

In the manuscript, we refer to the *precision* of single-point energy calculations and *accuracy* of the electric properties. Accuracy is related to the absolute difference with respect to the reference value [*e.g.*, RI-MP2 vs. CCSD(T)], whereas precision is used to indicate to which extent all energy calculations are similarly close to the reference energy. For instance, if an approximate method is systematically off by 50 units, it will be inaccurate but precise. If we were concerned about absolute energies, accuracy would be the only criterion considered. However, in order to obtain an accurate energy derivative (and, hence, an electric property) the approximate methods need to give precise energies, otherwise they will give a poor derivative. Numerical errors are the main leak of precision, because they do not tend to follow a systematic pattern. Hence, very accurate data might be sufficiently imprecise to generate large derivative errors. For this reason, the information on the energy accuracy, while very relevant for energy benchmark studies, it is irrelevant for response properties. In this section, *precision* errors associated to the numerical errors originated from the numerical derivative calculations are addressed using the mean absolute relative Romberg error (MARoE) [see manuscript].

In Table S10, we collect the MARoE and the ratio %MARoE of the Romberg error associated to each property for the canonical and accelerated MP2 methods. We shall stress here that the Romberg error is not equal to the numerical error, but constitutes an estimation of its order of magnitude.

Table S10: Mean Absolute Romberg Error (MARoE) and ratio between MARoE and the mean value (%MARoE) obtained using the canonical, accelerated and enhanced MP2 methods for molecules of the γ-NLO set. For the DLPNO-MP2-α method, the values of μ and α are computed analytically and therefore there is no associated RE. (*tight* and *vtight* cutoffs used for DLPNO and LNO, respectively)

		RI-MP2	RIJK-MP2	RIJCOSX2-MP2	LMP2	DLPNO-MP2	DLPNO-MP2-α	SCS-MP2	SOS-MP2	MP2
μ	MARoE	2.3E-07	1.1E-07	6.1E-07	1.9E-04	1.5E-04	-	1.4E-07	1.5E-07	2.3E-07
	%MARoE	0	0	0	0	0	-	0	0	0
α	MARoE	7.4E-03	1.2E-03	2.7E-02	1.2E-01	4.0E-01	-	4.8E-04	5.3E-04	1.5E-03
	%MARoE	0	0	0	0	0	-	0	0	0
β	MARoE	5.9E-01	2.1E-01	1.3E+00	2.9E+01	4.8E+01	2.4E-01	4.3E-01	4.4E-01	6.2E-01
	%MARoE	0	0	1	16	13	0	0	0	0
γ	MARoE	2.2E+03	4.4E+03	1.6E+04	3.2E+05	4.0E+05	5.8E+03	1.6E+03	1.9E+03	2.3E+03
	%MARoE	0	1	3	87	80	1	0	0	0

In Table S11, we collect the MARoE and %MARoE of CCSD and CCSD(T) methods and the corresponding approximations.

Table S11: Mean Absolute Romberg Error (MARoE) and ratio between MARoE and the mean value (%MARoE) obtained using the canonical and accelerated CCSD and CCSD(T) methods for molecules of γ-NLO set (*tight* and *vtight* cutoffs used for DLPNO and LNO, respectively).

		DLPNO-CCSD	DLPNO-CCSD(T0)	DLPNO-CCSD(T1)	LNO-CCSD	LNO-CCSD(T)	CCSD	CCSD(T)
μ	MARoE	3.5E-04	2.1E-04	1.7E-04	1.2E-04	1.5E-04	1.1E-04	1.5E-06
	%MARoE	0	0	0	0	0	0	0
α	MARoE	3.5E-01	6.0E-01	2.9E-01	2.1E-01	3.8E-01	2.5E-01	4.1E-03
	%MARoE	0	1	0	0	0	0	0
β	MARoE	1.5E+02	4.3E+01	2.6E+01	2.9E+01	2.7E+01	3.3E+01	1.4E+00
	%MARoE	61	24	17	13	13	15	1
γ	MARoE	3.2E+05	2.0E+05	1.8E+05	1.2E+05	1.6E+05	3.1E+04	2.5E+04
	%MARoE	48	60	41	32	25	5	6

4 Impact of the different convergence criteria on the accuracy of the localization schemes

Table S12: Performance of localized CCSD methods with respect to CCSD(T) references for molecules of the γ-NLO set. MV, MAE, RMSE, and MAX are in a.u.

		**DLPNO-CCSD**		**LNO-CCSD**			
		Normal	Tight	Normal	Tight	vTight	**CCSD**
μ	MV	7.2E-01	7.2E-01	7.2E-01	7.2E-01	7.2E-01	7.2E-01
	MARoE	4.8E-04	3.5E-04	5.0E-04	1.3E-04	1.2E-04	1.1E-04
	MAE	1.8E-02	2.1E-02	2.1E-02	2.1E-02	2.2E-02	2.2E-02
	RMSE	2.7E-02	3.1E-02	3.1E-02	3.1E-02	3.2E-02	3.4E-02
	MAX	7.1E-02	7.7E-02	8.8E-02	7.2E-02	8.2E-02	9.5E-02
	%MAE	3	3	3	3	3	3
α	MV	1.2E+02	1.1E+02	1.1E+02	1.1E+02	1.1E+02	1.1E+02
	MARoE	1.8E+00	3.5E-01	1.9E+00	5.4E-01	2.1E-01	2.5E-01
	MAE	1.7E+01	3.8E+00	6.8E+00	4.3E+00	3.4E+00	3.0E+00
	RMSE	7.1E+01	1.0E+01	2.2E+01	1.1E+01	8.3E+00	8.8E+00
	MAX	4.3E+02	6.1E+01	1.4E+02	5.5E+01	4.4E+01	5.9E+01
	%MAE	15	3	6	4	3	3
β	MV	2.7E+02	2.4E+02	4.7E+01	2.5E+02	2.2E+02	1.9E+02
	MARoE	2.4E+02	1.5E+02	4.4E+01	4.5E+01	2.9E+01	3.3E+01
	MAE	1.4E+02	1.3E+02	1.1E+02	1.2E+02	9.1E+01	5.1E+01
	RMSE	4.2E+02	2.9E+02	2.8E+02	3.1E+02	2.5E+02	1.3E+02
	MAX	1.5E+03	1.1E+03	1.1E+03	1.2E+03	9.2E+02	4.6E+02
	%MAE	103	91	77	88	66	37
γ	MV	1.4E+07	6.7E+05	1.1E+06	3.3E+05	3.8E+05	3.8E+05
	MARoE	1.4E+06	3.2E+05	9.7E+05	6.5E+05	1.2E+05	3.1E+04
	MAE	1.2E+07	4.3E+05	6.8E+05	2.0E+05	1.5E+05	8.4E+04
	RMSE	5.3E+07	9.7E+05	2.2E+07	6.3E+06	7.2E+05	3.7E+05
	MAX	3.0E+08	5.9E+06	2.2E+07	6.3E+06	5.3E+06	2.7E+06
	%MAE	2651	94	150	43	32	19

Table S13: Performance of localized CCSD(T) methods with respect to CCSD(T) references for molecules of the γ-NLO set. MV, MAE, RMSE, and MAX are in a.u.

		**DLPNO-CCSD(T0)**		**DLPNO-CCSD(T1)**		**LNO-CCSD(T)**		
		**Normal**	**Tight**	**Normal**	**Tight**	**Normal**	**Tight**	**vTight**
μ	MV	7.0E-01	7.0E-01	7.0E-01	7.0E-01	7.0E-01	7.0E-01	7.0E-01
	MARoE	3.5E-04	2.1E-04	4.1E-04	1.7E-04	3.2E-04	3.6E-04	1.5E-04
	MAE	6.3E-03	2.4E-03	8.5E-03	2.0E-03	6.6E-03	4.1E-03	2.3E-03
	RMSE	1.7E-02	3.9E-03	2.4E-02	5.1E-03	1.3E-02	7.0E-03	3.1E-03
	MAX	7.6E-02	1.3E-02	8.2E-02	2.0E-02	5.2E-02	3.0E-02	1.3E-02
	%MAE	1	0	1	0	1	0	0
α	MV	1.2E+02	1.1E+02	1.2E+02	1.2E+02	1.1E+02	1.2E+02	1.2E+02
	MARoE	1.3E+00	6.0E-01	4.9E-01	2.9E-01	3.1E+00	6.4E-01	3.8E-01
	MAE	1.2E+01	1.5E+00	2.9E+01	8.2E-01	7.3E+00	2.7E+00	1.2E+00
	RMSE	5.7E+01	4.2E+00	1.1E+02	2.1E+00	2.2E+01	7.7E+00	3.9E+00
	MAX	3.3E+02	2.0E+01	5.3E+02	1.0E+01	1.2E+02	4.0E+01	2.1E+01
	%MAE	10	1	25	1	6	2	1
β	MV	2.6E+02	1.8E+02	3.0E+02	1.5E+02	4.0E+02	3.7E+02	2.2E+02
	MARoE	1.3E+02	4.3E+01	2.2E+02	2.6E+01	1.6E+02	1.0E+02	2.7E+01
	MAE	1.4E+02	8.9E+01	2.0E+02	2.5E+01	3.2E+02	2.7E+02	9.2E+01
	RMSE	3.9E+02	2.9E+02	5.0E+02	4.8E+01	1.3E+03	7.6E+02	3.4E+02
	MAX	1.4E+03	1.3E+03	1.5E+03	1.9E+02	6.2E+03	3.0E+03	1.6E+02
	%MAE	102	65	142	18	233	195	67
γ	MV	1.0E+06	3.3E+05	1.7E+07	4.5E+05	1.6E+06	1.3E+06	6.3E+05
	MARoE	7.9E+06	2.0E+05	2.7E+05	1.8E+05	7.9E+06	6.6E+05	1.6E+05
	MAE	7.1E+05	1.8E+05	1.4E+07	1.5E+05	1.2E+06	1.2E+06	1.8E+05
	RMSE	3.0E+06	7.0E+05	6.3E+07	5.3E+05	4.9E+06	6.6E+06	1.0E+06
	MAX	1.9E+07	5.1E+06	3.7E+08	3.3E+06	3.3E+07	5.0E+07	7.7E+06
	%MAE	158	39	3020	33	257	265	41

Table S14: Performance of RI-MP2 methods with respect to CCSD(T) references for molecules of the γ-NLO set. MV, MAE, RMSE, and MAX are in a.u.

		**RI-MP2**	**RI-MP2:RIJK**	**RIJCOSX-MP2**	**RIJCOSX2-MP2**	**MP2**
μ	MV	7.2E-01	7.2E-01	7.2E-01	7.2E-01	7.2E-01
	MARoE	2.3E-07	1.1E-07	1.0E-05	6.1E-07	2.3E-07
	MAE	3.3E-02	3.3E-02	3.3E-02	3.3E-02	3.2E-02
	RMSE	4.7E-02	4.7E-02	4.8E-02	4.7E-02	4.7E-02
	MAX	1.3E-01	1.3E-01	1.3E-01	1.3E-01	1.3E-01
	%MAE	5	5	5	5	5
α	MV	1.2E+02	1.2E+02	1.2E+02	1.2E+02	1.2E+02
	MARoE	7.4E-03	1.2E-03	2.1E-02	2.7E-02	1.5E-03
	MAE	3.7E+00	3.7E+00	5.0E+00	4.1E+00	3.7E+00
	RMSE	7.2E+00	7.3E+00	1.4E+01	9.1E+00	7.3E+00
	MAX	3.1E+01	3.1E+01	9.1E+01	4.6E+01	3.1E+01
	%MAE	3	3	4	4	3
β	MV	2.4E+02	2.4E+02	2.2E+02	2.4E+02	2.4E+02
	MARoE	5.9E-01	2.1E-01	1.7E+01	1.3E+00	6.2E-01
	MAE	1.1E+02	1.1E+02	9.2E+01	1.1E+02	1.1E+02
	RMSE	2.6E+02	2.7E+02	2.2E+02	2.8E+02	2.6E+02
	MAX	8.3E+02	9.0E+02	8.4E+02	1.0E+03	8.9E+02
	%MAE	77	80	67	81	78
γ	MV	6.0E+05	5.7E+05	5.6E+05	5.6E+05	5.2E+05
	MARoE	2.2E+03	4.4E+03	1.3E+04	1.6E+04	2.3E+03
	MAE	1.5E+05	1.3E+05	1.2E+05	1.1E+05	7.7E+04
	RMSE	6.0E+05	4.4E+05	4.2E+05	3.4E+05	2.4E+05
	MAX	3.4E+06	2.3E+06	2.3E+06	1.6E+06	1.2E+06
	%MAE	34	28	27	25	17

Table S15: Performance of localized MP2 methods with respect to CCSD(T) references for molecules of the γ-NLO set. MV, MAE, RMSE, and MAX are in a.u.

			**LMP2**		**DLPNO-MP2**		**DLPNO-MP2-α**	
		**Normal**	**Tight**	**vTight**	**Normal**	**Tight**	**Normal**	**Tight**
μ	MV	7.2E-01	7.2E-01	7.2E-01	7.2E-01	7.2E-01	7.2E-01	7.2E-01
	MARoE	1.9E-04	1.4E-05	1.9E-04	2.1E-04	1.5E-04	x	x
	MAE	3.4E-02	3.3E-02	3.2E-02	3.2E-02	3.2E-02	3.3E-02	3.3E-02
	RMSE	4.9E-02	4.8E-02	4.7E-02	4.7E-02	4.6E-02	4.7E-02	4.7E-02
	MAX	1.4E-01	1.3E-01	1.2E-01	1.3E-01	1.3E-01	1.3E-01	1.3E-01
	%MAE	5	5	5	5	5	5	5
α	MV	1.2E+02	1.2E+02	1.2E+02	1.2E+02	1.2E+02	1.2E+02	1.2E+02
	MARoE	2.0E+00	3.0E-01	1.2E-01	6.0E-01	4.0E-01	x	x
	MAE	4.4E+00	3.8E+00	3.4E+00	4.1E+00	4.3E+00	3.5E+00	3.6E+00
	RMSE	9.9E+00	7.8E+00	6.7E+00	9.2E+00	9.3E+00	6.8E+00	7.1E+00
	MAX	4.5E+01	3.4E+01	2.8E+01	4.8E+01	5.0E+01	2.9E+01	3.0E+01
	%MAE	4	3	3	3	4	3	3
β	MV	3.0E+02	1.5E+02	1.8E+02	2.2E+02	2.7E+02	2.3E+02	2.4E+02
	MARoE	4.2E+02	1.3E+01	2.9E+01	1.3E+02	4.8E+01	4.7E-01	2.4E-01
	MAE	1.3E+02	6.1E+01	5.7E+01	9.8E+01	2.5E+02	1.0E+02	1.1E+02
	RMSE	4.2E+02	1.3E+02	1.2E+02	2.4E+02	6.4E+02	2.4E+02	2.6E+02
	MAX	1.7E+03	4.4E+02	3.5E+02	1.1E+03	2.4E+03	8.2E+02	8.7E+02
	%MAE	128	44	41	71	182	74	78
γ	MV	8.9E+05	1.2E+06	3.6E+05	4.2E+05	5.0E+05	5.7E+05	5.5E+05
	MARoE	6.0E+06	8.5E+05	3.2E+05	6.2E+05	4.0E+05	5.6E+03	5.8E+03
	MAE	4.7E+05	7.5E+05	2.6E+05	2.1E+05	1.5E+05	1.3E+05	1.0E+05
	RMSE	2.1E+06	4.2E+06	1.2E+06	7.4E+05	3.8E+05	4.5E+05	3.7E+05
	MAX	1.5E+07	3.2E+07	8.9E+06	3.7E+06	1.9E+06	2.2E+06	2.1E+06
	%MAE	103	166	58	46	32	28	23

Table S16: Performance of DLPNO-MP2 method (α computed as second derivative of the energy) with respect to DLPNO-MP2-α method (that provides analytical values of μ and α, the latter of which is employed to compute the energy derivatives that give rise to β and γ).

		DLPNO-MP2
μ	MV	7.2E-01
	MAE	6.1E-03
	RMSE	1.4E-03
	MAX	4.9E-03
	%MAE	0
α	MV	1.2E+02
	MAE	1.0E+00
	RMSE	3.1E+00
	MAX	2.0E+01
	%MAE	1
β	MV	3.7E+02
	MAE	1.2E+02
	RMSE	2.5E+02
	MAX	8.4E+02
	%MAE	49
γ	MV	5.0E+05
	MAE	2.2E+05
	RMSE	5.7E+05
	MAX	2.7E+06
	%MAE	40

5 Performances of accelerated methods on the β-Set

Table S17: Performance of DLPNO-CCSD(T1), MP2, RI-MP2 and CCSD methods for the β-NLO set with respect to the reference CCSD(T). MV, MAE, RMSE, and MAX are in a.u.

		**DLPNO-CCSD(T1)**	**RI-MP2**	**MP2**	**CCSD**
	MV	3.3E+00	3.4E+00	3.4E+00	3.3E+00
	MARoE	1.9E-03	2.8E-05	1.7E-08	4.9E-06
	%MARoE	0	0	0	0
μ	MAE	2.3E-02	1.0E-01	1.0E-01	5.6E-02
	RMSE	3.1E-02	1.2E-01	1.2E-01	6.7E-02
	MAX	9.8E-02	2.9E-01	2.9E-01	1.4E-01
	%MAE	1	3	3	2
	MV	3.5E+02	3.6E+02	3.6E+02	3.3E+02
	MARoE	6.0E+00	6.0E-02	9.8E-05	7.3E-03
	%MARoE	2	0	0	0
α	MAE	1.0E+01	1.7E+01	1.7E+01	1.3E+01
	RMSE	2.3E+01	2.7E+01	2.7E+01	1.7E+01
	MAX	1.2E+02	9.5E+01	9.4E+01	5.1E+01
	%MAE	3	5	5	4
	MV	1.0E+04	1.2E+04	1.2E+04	9.2E+03
	MARoE	6.6E+03	3.0E+02	4.7E-01	1.8E+01
	%MARoE	65	0	0	0
β	MAE	1.5E+03	2.8E+03	2.8E+03	7.7E+02
	RMSE	2.4E+03	4.5E+03	4.5E+03	1.2E+03
	MAX	6.8E+03	1.3E+04	1.3E+04	4.5E+03
	%MAE	15	28	27	8
	MV	3.5E+06	2.9E+06	2.9E+06	2.3E+06
	MARoE	1.1E+05	1.6E+03	5.6E+04	7.4E+04
	%MARoE	3	0	2	3
γ	MAE	1.6E+06	5.6E+05	5.2E+05	3.9E+05
	RMSE	3.7E+06	9.8E+05	8.7E+05	7.0E+05
	MAX	1.7E+07	3.1E+06	2.9E+06	3.2E+06
	%MAE	51	21	19	14

6 Performances of the accelereted methods merging the β-NLO and γ-NLO-B sets

Table S18: Performance of DLPNO-CCSD(T1), MP2, RI-MP2, and CCSD methods for β with respect to the reference CCSD(T), merging the β-NLO and γ-NLO-B sets. MV, MAE, RMSE, and MAX are in a.u.

		**DLPNO-CCSD(T1)**	**RI-MP2**	**MP2**	**CCSD**
	MV	7.3E+03	8.6E+03	8.5E+03	6.6E+03
	MAE	1.1E+03	2.0E+03	2.0E+03	5.6E+02
β	RMSE	2.1E+03	3.8E+03	3.8E+03	1.0E+03
	MAX	6.8E+03	1.3E+04	1.3E+04	4.5E+03
	%MAE	16	28	28	8

7 Cartesian coordinates for the molecules of the γ-NLO and β-NLO sets

All cartesian coordinates can be accessed at the following DOI: 10.19061/iochem-bd-4-53 onto the IOCHEM-BD platform (www.iochem-bd.org) to facilitate data exchange and dissemination, according to the FAIR principles of OpenData sharing.
